# Telephone Survey Versus Panel Survey Samples Assessing Knowledge, Attitudes and Behavior Regarding Animal Welfare in the Red Meat Industry in Australia

**DOI:** 10.3389/fpsyg.2021.581928

**Published:** 2021-04-08

**Authors:** Lauren M. Hemsworth, Maxine Rice, Paul H. Hemsworth, Grahame J. Coleman

**Affiliations:** Animal Welfare Science Centre, Faculty of Veterinary and Agricultural Sciences, University of Melbourne, Parkville, VIC, Australia

**Keywords:** public attitudes, behavior, random digit dialing telephone survey, probability internet panel survey, animal use, animal welfare, red meat industry

## Abstract

Surveys are used extensively in social research and, despite a lack of conclusive evidence of their ‘representativeness,’ probability internet panel (PIP) surveys are being increasingly used to make inferences about knowledge, attitude and behavior in the general population regarding a range of socially relevant issues. A large-scale survey of Australian public attitudes and behavior toward the red meat industry was undertaken. Samples were obtained using a random digit dialing telephone survey (Computer-Assisted Telephone Interviewing-CATI, *n* = 502 respondents) and a PIP survey (PANEL, *n* = 530 respondents) to examine differences between the two samples regarding attitudes and behavior relating to livestock use and welfare. There was little difference in demographics between the CATI and the PANEL surveys apart from highest level of education. However, there were differences between the two samples in both attitudes and behavior toward the red meat industry after controlling for education levels. The PANEL respondents gave generally more conservative responses than did the CATI respondents in the sense that they were more positive toward the livestock industries and animal welfare within these industries. Differences were also found between the respondents of the two samples regarding behavior that relates to the red meat industry, both community and consumer behavior. PANEL respondents were less engaged in community behaviors performed in opposition of the red meat industry when compared with the CATI sample. The majority of CATI and PANEL respondents were red meat eaters and there was no difference between respondents of the two samples in relation to red meat consumption, however, there were fewer vegetarians and vegans in the PANEL survey. Possible reasons for the observed differences are discussed, however, a definitive answer will depend on further research to identify the specific psychological factors that differ between samples derived from different survey methodologies.

## Introduction

Social research relies heavily on surveys. High marginal costs and low response rates have reduced the viability of random telephone surveys (RDD; random digit dialing) whilst increasing the viability of surveys delivered online (internet surveys) (Berrens et al., 2003; Li et al., 2004; Ansolabehere and Schaffner, 2014). Whilst telephone surveys reportedly generated higher participant response rates than online or mail delivery (Yu and Cooper, 1983) and data quality that was comparable to that obtained from face-to-face interviews (Groves and Kahn, 1979), it has become increasingly difficult to maintain participant response rates and, as a result, the costs of telephone data collection has risen considerably (Lavrakas, 1997; Holbrook et al., 2007; Chang and Krosnick, 2009). Internet surveys offer several advantages, including low marginal cost per completed response, an ability to provide respondents with large quantities of information, speed and the elimination of interviewer bias (see review by Couper, 2000; Berrens et al., 2003). Ansolabehere and Schaffner (2014) compared internet, mail and telephone surveys and found response rates of 42.9, 21.1, and 19.5%, respectively, with completion times of 8.9, 11.8 and 14.3 min, respectively. A comparison of the internet and telephone attitude responses to a range of political issues showed that they were quite similar and that the main differences were in cost (telephone more expensive than internet), response rates and completion time. Given the increasing access to the internet, these cost differences are likely to have increased in the 10 years since the Ansolabehere and Schaffner (2014) study was carried out. More recently, Lee et al. (2019) conducted a comparison between a computer web survey, a smartphone web survey and a computer-assisted telephone interviewing (CATI) survey on student time use, opinions on university life and courses, health status, online access for health information, and demographic information. The CATI survey achieved the highest response rate, but also the highest cost and the longest completion time.

There are two primary methodologies that have been employed by commercial survey companies to conduct internet surveys; non-probability samples and probability samples (Couper, 2000; Chang and Krosnick, 2009). Non-probability sampling involves internet surveys of volunteers that are not recruited using conventional random sampling methods, i.e., do not have known non-zero probabilities of selection. Non-probability samples involve ‘self-selection’ and employ a range of methods to recruit survey participants including advertisements placed on websites inviting people to sign up to do regular surveys and email invitations that are widely distributed in ways designed to yield responses from heterogeneous population subgroups with internet access (Yeager et al., 2011). A comparison of responses to a health care survey from non-probability internet samples and probability samples using an RDD telephone survey have shown that there are differences in responses even when the internet sample was propensity weighted (Schonlau et al., 2004). About 80% of questions were responded to differently, however, there was no systematic reason for the differences in responses. One unexplained outcome was that “web survey responses were significantly more likely to agree with RDD responses when the question asked about the respondent’s personal health (9 times more likely), was a factual question (9 times more likely), and only had two as opposed to multiple response categories (17 times more likely)” (Schonlau et al., 2004).

Probability internet panel (PIP) sampling most commonly involves the use of a pre-existing online panel sample, whose panel lists were originally recruited to the panel using random sampling methods such as RDD telephone surveys. Initial telephone interviews are used to collect background information and invite eligible people to join the online panel. The aim is to obtain a probability sample of internet users who, following agreement to join the online panel, are sent email requests to participate in internet surveys (Couper, 2000), usually for a monetary or points reward. Studies involving this type of probability internet survey are largely reported as random participant recruitment (Duncan, 2015). Online panels are being increasingly used in social science to recruit participants for community surveys and questionnaires, where inferences are often made about the general population based on the findings of a ‘random’ sample of participants. At present, however, there remains little conclusive evidence demonstrating that internet surveys based on probability sampling (i.e., ‘random’ online panel samples) are in fact representative of the general population, both in terms of demographic and psychological aspects. Despite commercial online panels largely employing random sampling methods to recruit panel lists and the opportunity to use stratification to control for demographic factors, it has still been suggested that there remain substantial differences between the online population (PIP survey) and the general population (RDD telephone survey) with regards to the substantive variables of interest such as attitudes and behaviors (see review by Couper, 2000). Previous research investigating differences between the two populations has been inconclusive, with some studies from countries other than Australia finding differences between respondents from PIP surveys and RDD telephone surveys (for example, United States: Flemming and Sonner, 1999; United Kingdom: Erens et al., 2014; South Korea: Lee et al., 2015) and others finding no difference (for example, United States: Berrens et al., 2003; Li et al., 2004).

In those studies where differences were found, the survey content was related to co-morbidities associated with gambling (Lee et al., 2015) and politics and voting (Flemming and Sonner, 1999). In the lead up to an election, Flemming and Sonner (1999) compared respondents from a RDD telephone survey and a weighted (by sex and education level) PIP survey. Important differences were found between the samples on a variety of attitudinal items, including interest in the election, attitudes toward impeachment and the role of national issues in congressional voting. The authors conclude “there were no predictable patterns to the success or failure of the internet surveys. Respondents … were not consistently more conservative or liberal than those in nationwide telephone surveys, nor were they more optimistic or pessimistic”. This “raises important questions about the utility of internet polls to replace traditional telephone survey practices” (Flemming and Sonner, 1999, 13). More recently, Lee et al. (2015) used a stratified sampling method to ensure age X gender quotas and a *post hoc* weighting method to compensate for age X gender sampling deviations from the population. Despite this, they found significant differences between RDD and online samples on tobacco use, drug and alcohol problems, happiness level and mental health problems, with the higher pathologies occurring in the online sample. In those studies where differences were not found, the surveys targeted climate change (Berrens et al., 2003) and global warming (Li et al., 2004).

There appears to have been a tendency recently for researchers investigating public attitudes to farm animal welfare to utilize PIP samples (for example, Worsley et al., 2015; Malek et al., 2018; Bir et al., 2019; Connor and Cowan, 2020; Jackson et al., 2020) and few examples where RDD telephone samples have been used. There have been no investigations into whether PIP samples and RDD telephone samples yield similar results when targeting public perceptions of farm animal welfare. It is important to know whether the substantive results from PIP surveys represent the population from which the samples were drawn or whether there are systematic biases. Such research may provide a cost-effective alternative to RDD telephone surveys in animal welfare research.

The data analyzed in this paper were derived from a current research project examining public and producer attitudes and knowledge toward sheep and beef cattle welfare in Australia. Literature suggests that public attitudes to animal welfare impact the livestock industries not just by influencing purchasing of animal products, but also by underpinning a range of community behaviors in opposition of the livestock industry, such as signing petitions, donating money to welfare organizations and speaking to colleagues about animal welfare issues (Coleman et al., 2017; Coleman, 2018). Public attitudes toward the livestock industries, livestock animal welfare and trust in the livestock industries were related to meat consumption as well as behaviors that people engage in that may impact on the pork industry (i.e., community behaviors). Regression analyses demonstrated that these variables accounted for significant proportions of the variance in both pork consumption and in community behavior (Coleman et al., 2017; Coleman, 2018). These behaviors and the attitudes driving them can have a considerable influence on how Governments either react to publicized ‘animal welfare events’ or regulate contentious management practices in industry. Our current research project extended this into the investigation of these relationships in the Australian red meat industry. In addition to the range of general attitudes, knowledge of husbandry practices and trust of livestock industry people and information sources, we also assessed in our current research project, behavior-specific attitudes utilizing the Theory of Planned Behavior (TPB: Ajzen, 1988). These latter variables comprise attitudes to the specific behavior of interest, normative beliefs (beliefs about the expectations of salient others) and control beliefs (beliefs about personal capacity to perform the behavior). Because of the substantial cost associated with RDD telephone surveys, it was decided that two samples drawn from the Australian public were to be obtained; one using a RDD telephone survey (Computer Assisted Telephone Interview – CATI) which was high cost and the other a PIP survey (PANEL, based on probability samples) which was relatively low cost. The aim in this paper therefore was to determine whether there were differences between the two survey samples in attitudes and behavior relating to animal welfare in the Australian red meat industry. Specifically, two broad questions were addressed: were the two samples similar in terms of attitudes and behavior and were the relationships between attitudes and behavior similar for the two samples?

## Materials and Methods

### Development and Structure of Questionnaire

A questionnaire was developed using an iterative process that began with questionnaires that had been developed by the Animal Welfare Science Centre (AWSC) for livestock industries including the pork, egg and red meat industries (see Coleman and Toukhsati, 2006; Coleman et al., 2016, 2017). These questionnaires were adapted to target attitudes toward the red meat industry, animal welfare and husbandry practices. The questionnaires also assessed the participant’s knowledge of farm animals and farm animal welfare, the frequency with which they accessed information on animal welfare, the source of information they most frequently used and trusted and the extent to which they engaged in community behaviors such as calling talk-back radio and writing to a politician to express dissatisfaction toward the red meat industry. The sections of the questionnaire are reported in Table 1.

**TABLE 1:** Structure of the questionnaire.

Section	Information gathered
Demographics	Age, Gender, education, location, red meat consumption,
Animal welfare	General attitudes toward animal welfare, trust of people involved in farm animal production, normative and control beliefs in relation to animal welfare
Knowledge of farm animals and farm animal welfare	Perceived and actual knowledge of beef cattle and sheep production practices (e.g., curfew, mulesing, castration, etc.)
Attitudes toward red meat farming practices	Approval of red meat farming practices, importance of social contact, fresh air, exercise, etc., concern about transport conditions.
Behavior in relation to farm animal welfare	Animal rights group membership, community behaviors, sources of animal welfare information, discussions about animal welfare

### Participant Recruitment and Collection of Data

Human ethics approval was obtained from The University of Melbourne’s Human Ethics Advisory Group (Ethics ID: 1750676.3). Before the questionnaire was undertaken, respondents were given a plain language statement (i.e., an explanatory statement outlining the research aims), advised that participation was entirely voluntary and that they could withdraw at any time if so desired and consent was sought. I-View, a specialized market and social research data collection agency, were contracted to deliver the questionnaire to 1000 members of the Australian general public, using two ‘random’ participant recruitment methods; 502 participants were surveyed using a RDD telephone recruitment (CATI) and a further 530 participants from a PIP (PANEL). Both samples were subjected to a 50:50 gender split and an age distribution consistent with Australian census data. The average duration of the CATI survey was 33.3 min and the response rate was 15%. For the PANEL survey, the median duration was 19 min (median used because of occasional outliers caused by respondents being logged on for very long periods) and the response rate estimated to be 10% based on the number of respondents emailed who clicked on the survey link. Data collection for CATI commenced on 21st March 2018 and was completed on the 16th April 2018, while the PANEL commenced on 29th March 2018 and was completed on the 16th April 2018.

CATI involved dialing random fixed-line (*n* = 246) and mobile telephone numbers (*n* = 256) and inviting potential respondents to complete the questionnaire by telephone. Recent research (Kennedy et al., 2018) has suggested that using both fixed-line and mobile telephone numbers provides the most demographically representative sample and does not bias data collected. In each call, the consultant requested the youngest male in the household (over the age of 18 years) in order to counteract the expected bias for older female participants commonly encountered in telephone surveys. This was used as a first step after which any available person was interviewed if they met the quota requirements. The PANEL “MyView” was originally recruited by recruitment service providers, conducting email marketing campaigns, social media marketing campaigns and traditional marketing campaigns using a points-rewards based system for incentives where participants are awarded points by completing surveys. All panelists undergo a comprehensive validation process to ensure no duplication and are screened for IP address within Australia and age groups over 14 years old. Email confirmations are also used to ensure that the email is valid and belongs to the person that completed the recruitment questionnaire. MyView panel participants over the age of 18 were invited via email to participate in the current survey for a payment of 300 points (AUD $3.00). If a respondent accessed the survey and was in an age or gender group that had met the quota requirements, they were screened out of the survey. The survey was then displayed on their Panel dashboard and appeared as a notification on their mobile device if they had downloaded the application.

### Statistical Analysis

Statistical analyses were performed using the statistical package SPSS 25.0 (SPSS Inc., Chicago, IL, United States). The attitude, beliefs and trust sections of the questionnaire (sections B and D of the questionnaire, see Table 1) data were analyzed using Principal Components Analysis (PCA), followed by either a Varimax or an Oblimin rotation, to identify commonalities amongst the questionnaire items (see Table 2). The suitability of the data for the analysis was assessed using criteria outlined by Pallant (2013); the correlation matrix coefficients were all above the required 0.3, the Kaiser-Meyer-Olkin (KMO) values exceeded the recommended value of 0.6, and Bartlett’s Test of Sphericity reached statistical significance. Items that were established as belonging to a common underlying component were then summed to produce a composite score for that component. Before conducting the PCAs, items were recoded where appropriate so that high scores reflected positive attitudes, high trust, etc. Scale reliabilities were measured using Cronbach’s α coefficients with an α > = 0.70 as the criterion for acceptable reliability (DeVellis, 2003). Items were included in a scale if their loading on the relevant component exceeded 0.33 (Tabachnick and Fidell, 2012) and if, on the basis of face validity, they could be summarized by just one construct.

**TABLE 2:** Components from the questionnaire grouped into composite scores, a high score indicating a positive attitude or strong agreement to the statements.

Topic	Assigned attitude component label	Cronbach’s Alpha	Questionnaire item
The meaning of animal welfare	Humane treatment	0.82	Humane treatment of animals
			Preventing animal cruelty
			Protecting the rights of animals
	Best practice handling	0.78	Farmers and farm animal handlers using best practice
			Farmers and farm animal handlers caring for their animals
	Caring for and balancing the needs of pets and people	0.57	Caring for our pets Balancing the needs of animals and people
Acceptability of animal uses	Red meat attributes	0.81	I believe beef and lamb are healthy foods
			It is appropriate to use sheep and beef cattle to produce food for humans
			Sheep and beef cattle farming is environmentally sustainable
			Sheep and beef cattle are raised in a humane and animal friendly manner
	Red meat animal rights	0.69	Sheep and beef cattle have the same right to life as domestic animals
			Sheep and beef cattle have the same feelings as domestic animals
Behavioral beliefs	Public engagement beliefs	0.89	I think it is important to lobby governments to improve the welfare of farm animals
			I should encourage my friends to support animal welfare causes
			It is important for me to be actively involved in the promotion of farm animal welfare
			It is important for me to encourage family and friends to be actively involved in the promotion of animal welfare
Normative beliefs	Negative normative beliefs	0.74	The welfare of farm animals is not something that my partner/family would expect me to consider when making meat shopping choices
			Lobbying the government to improve the welfare of farm animals is not something my partner/family would expect me to do
			My partner/family would not expect me to encourage my family and friends to be actively involved in the promotion of animal welfare
	Positive normative beliefs	0.78	My partner/family would expect me to buy lamb and beef that is produced with good animal welfare practices
			My partner/family would expect me to encourage my friends to support animal welfare causes
			My partner/family would expect me to be actively involved in the promotion of farm animal welfare
Control beliefs	Difficult to act	0.48	I find it takes too much effort to buy beef and lamb that is produced with good animal welfare practices.
			I would find it too difficult to lobby the government to improve the welfare of farm animals
	Easy to act	0.75	I can easily encourage my friends to support animal welfare causes
			I can easily be involved actively in the promotion of farm animal welfare
Trust of livestock industry people	Trust	0.92	I trust farmers to properly care for their sheep and beef cattle
			I trust farm animal handlers to properly care for their sheep and beef cattle
			I trust those responsible for transporting sheep and beef cattle by land to properly care for them
			I trust abattoir workers who work with sheep and beef cattle to properly care for them and use humane slaughter methods
Attitudes toward red meat farming practices	Approval of husbandry practices	0.89	Mulesing
			Crutching
			Dehorning
			Pre-slaughter stunning
			Curfew
			Tail docking
			Ear tagging
			Hot iron branding
			Castration
			Feedlotting
			Spaying
Importance of farming attributes	General welfare	0.95	Social contact with animals of the same species
			Contact with their young
			Shelter
			Access to water
			Freedom to roam outdoors
			Good nutrition
			Regular exercise
			Fresh air
			Protection from predators
			Pain relief during painful husbandry procedures
	Medication	0.8	Medications (i.e., antibiotics) for health
			Vaccinations for health
Comfort of beef cattle	Land beef transport conditions	0.94	Space per animal
			Provision of food and water
			Ventilation
			Journey length
			Road/truck conditions (e.g., sound, vibration, braking levels
			Loading of animals onto vehicles (e.g., use of handling aids, human handling)
	Sea beef transport conditions	0.96	Space per animal
			Provision of food and water
			Ventilation
			Journey length
			Boat conditions (e.g., sounds, vibration, unsteady ground)
			Loading of animals onto boats (e.g., use of handling aids, human handling)
Comfort of sheep	Land sheep transport conditions	0.96	Space per animal
			Provision of food and water
			Ventilation
			Journey length
			Road/truck conditions (e.g., sound, vibration, braking levels
			Loading of animals onto vehicles (e.g., use of handling aids, human handling)
	Sea sheep transport conditions	0.97	Space per animal
			Provision of food and water
			Ventilation
			Journey length
			Boat conditions (e.g., sounds, vibration, unsteady ground)
			Loading of animals onto boats (e.g., use of handling aids, human handling)
Accessing information	Commercial media	0.79	Government advertisements/promotions
			Celebrity chef/cook
			Industry bodies
			Supermarkets (e.g., Coles, Woolworths, IGA)
			Labels (product labels)
	Social and internet media	0.8	Internet
			Friends, relatives or colleagues
			Animal welfare organizations e.g., RSPCA
			Social network sites, related social media (e.g., Facebook, YouTube, Twitter, blogs)
	Conventional media	0.75	Television (e.g., TV news, documentaries)
			Radio
			Print media (e.g., magazines, newspapers, scientific papers)
Trust of information sources	Trust social and internet media	0.84	Television (e.g., TV news, documentaries)
			Radio
			Internet
			Print media (e.g., magazines, newspapers, scientific papers)
			Friends, relatives or colleagues
			Animal welfare organizations e.g., RSPCA
			Social network sites, related social media (e.g., Facebook, YouTube, Twitter, blogs)
	Trust conventional media	0.82	Government advertisements/promotions
			Industry bodies
			Supermarkets (e.g., Coles, Woolworths, IGA)
			Labels (product labels)
			Celebrity chef/cook

A summary of the details of the component structures are reported in Table 2, and most Cronbach’s α coefficients exceeded 0.7 with the exception of “Caring for and balancing the needs of pets and people” and “Easy to act” (0.57 and 0.48, respectively). In both cases the decision was made to retain the component because their component groupings showed good face validity and only two items comprised the composite score which is known to reduce the magnitude of Cronbach’s α coefficients (Nunnally et al., 1967).

Perceived knowledge was measured by asking the respondent “How much do you feel you know about beef cattle and sheep production?”. In addition, actual knowledge was assessed through a series of 13 multiple choice questions in relation to some common farming practices (e.g., mulesing, de-horning, castration, curfew, pre-slaughter stun, etc.). Respondents were then given a score (knowledge score) based on the proportion of correctly answered questions.

Community behavior was measured by the sum of the self-reported occurrences with which respondents said that they had engaged in acts such as calling talk-back radio, writing to newspapers and writing to politicians to express dissatisfaction with the red meat industry. Consumption of beef and lamb was measured by single items asking for the frequency of consumption of each.

Analyses of the demographic frequencies were carried out using Pearson χ^2^ tests of independence. Multivariate analysis of covariance (MANCOVA) with education as a covariate were conducted to compare the CATI and the PANEL samples on all composite variables. Independent 2-tailed *t*-tests with education as a covariate were then conducted on each of the composite variables separately to compare the responses of the CATI and the PANEL respondents. Correlations between the composite variables identified from the PCA on the attitudes, beliefs and trust items, perceived and actual knowledge, self-reported meat consumption, and community behaviors were conducted using Pearson product moment correlations. Separate stepwise multiple linear regressions were used to identify those variables that predicted each of the behaviors of interest – self-reported beef consumption, self -reported lamb consumption and community behaviors.

## Results

### Differences Between CATI and PANEL Samples: Demographics

Comparisons of CATI and PANEL respondents’ sociodemographic characteristics are reported in Table 3. The two survey samples are reasonably consistent with the most recent census data from the Australian Bureau of Statistics [ABS], 2016) and respondents from both surveys resided in all states and territories of Australia. There were no significant differences between the two samples regarding respondents’ geographical location (χ^2^_6_ = 14.69, *p* > 0.05), 50:50 gender split (χ^2^_2_ = 2.33, *p* > 0.05), or age distribution (χ^2^_5_ = 3.46, *p* > 0.05), with the 18–24 age group under-represented in both the CATI and PANEL samples (Table 3).

**TABLE 3:** Chi square comparison of CATI (*n* = 502) and PANEL (*n* = 530) respondents’ sociodemographic characteristics.

			CATI		PANEL		Census
			Count	%	Count	%	%
χ^2^_6_ = 6.10, *p* > 0.05	State/Territory	Victoria	137	28	135	26	24
		New South Wales	137	28	177	34	29
		Queensland	109	22	108	21	22
		South Australia	41	8	40	8	8
		Western Australia	50	10	49	9	12
		Tasmania	14	3	10	2	3
		Australian Capital Territory	10	2	6	1	2
χ^2^_2_ = 2.33, *p* > 0.05	Are you…?	Male	231	46	263	50	49
		Female	270	54	267	50	51
		Other	1	0	0	0	0
χ^2^_5_ = 3.46, *p* > 0.05	Which of these age groups are you in?	18–24	48	10	36	7	12
		25–34	75	15	78	15	19
		35–44	84	17	99	19	17
		45–54	96	19	106	20	17
		55–64	94	19	106	20	15
		65 +	105	21	105	20	20
χ^2^_3_ = 11.04, *p* < 0.05	What is your highest level of education?	No Formal Schooling	0	29	0	29.2	42.3
		Primary School	9		6		
		Secondary School	135		149		
		Technical or further educational institution (including TAFE College)	116	24.7	168	32	28.1
		University or other higher educational institution	230	46.3	204	38.8	27.3
χ^2^_3_ = 21.48, *p* < 0.05	How often do you do the grocery shopping for your household?	Rarely	48	10	21	4	
		Sometimes	86	17	69	13	
		Mostly	120	24	121	23	
		Always	247	49	319	60	
χ^2^_2_ = 6.98, *p* < 0.05	Would you describe yourself primarily as a…?	Meat and vegetable eater (A person who eats a variety of foods including red and white meat)	449	89	498	94	
		Vegetarian (A vegetarian is a person who does not eat red or white meat, including fish, but eats eggs and dairy products)	42	8	25	5	
		Vegan (A vegan is a person who eats no animal products at all)	11	2	7	1	

*Data for the Northern Territory and No formal schooling were not included in the analysis because of expected frequencies < 5. Census data of the Australian Bureau of Statistics [ABS] (2016) is also presented for geographical location, gender, age distribution and level of education.*

With regard to respondents’ highest level of education, there were significant differences between the CATI and PANEL samples (χ^2^_3_ = 11.04, *p* < 0.05; Table 3). The CATI sample had fewer technical and school leaver educated respondents than did the PANEL sample (χ^2^_1_ = 9.52, *p* < 0.05), but the other categories were not significantly different between the two samples. Numerically, the census percentages for technical and further education percentages were midway between the those for the two samples, while the census percentages for university or other higher education were below those for the two samples.

There was a significant difference between the samples in terms of who performs the household shopping (Table 3), with the CATI sample containing more respondents who did the shopping less frequently than the PANEL respondents (χ^2^_3_ = 21.48, *p* < 0.05). Despite the differences between the samples, most respondents from both samples were responsible for shopping in their households.

In relation to meat consumption, while most respondents from both samples were meat eaters, there was a significant difference between the samples in number of vegetarians and vegans, with fewer vegetarians and vegans in the PANEL sample (χ^2^_2_ = 6.98, *p* < 0.05). The vegetarian/vegan sample is relatively small, particularly in the PANEL survey (5 vs. 8%) and thus caution is required in interpreting this difference between the two samples.

### Differences Between CATI and PANEL Samples: Composite Variables and Behavioral Variables

Because the two survey samples differed with regard to education level, a MANCOVA with education as a covariate was used to compare the CATI and the PANEL sample on the composite variables. There was a significant effect for education (*F*_43_,_987_ = 2.66, *p* < 0.01). Following the MANCOVA, univariate tests were performed on each of the composite variables. Comparison of the CATI sample with the PANEL sample on the composite variables showed that the PANEL respondents gave generally more conservative responses than did the respondents from the CATI survey (*F*_43_,_987_ = 8.85, *p* < 0.01), in the sense that they were more positive toward the livestock industries and animal welfare within these industries (Table 4). The multivariable effect size for education was substantially smaller than that for sample type (Partial ^2^η = 0.10 and 0.28, respectively). The effect sizes (Cohen, 2016) of the significant univariate differences that were observed ranged from very small (< 0.2) to those in the small to medium range (> 0.2 but < 0.5). The PANEL sample also reported greater perceived knowledge (but not actual knowledge) of livestock production and were less engaged in communication activities and community behaviors.

**TABLE 4:** Independent 2-tailed *t*-tests (df = 1029) comparing the responses of the CATI and the PANEL respondents with education as a covariate.

			Adjusted Mean				
	t	Sig.	CATI	PANEL	Mean Difference (CATI-PANEL	Cohen’s D	Interpretation
**Animal welfare humane**	2.34	0.02	**4.41**	**4.29**	**0.12**	0.15	CATI respondents have a greater belief that animal welfare involves humane animal care/treatment
Animal welfare handling	1.54	0.12	4.28	4.20	0.08	0.09	No significant difference in CATI and PANEL respondents’ belief that animal welfare involves appropriate animal handling
**Animal welfare people animals**	2.71	0.01	**4.07**	**3.92**	**0.15**	0.17	CATI respondents have a greater belief that animal welfare involves a positive human-animal relationship.
**Red meat attributes**	−2.18	0.03	**3.65**	**3.77**	−**0.12**	−0.14	PANEL respondents have a more positive attitude toward red meat attributes, regarding human health, environmental impact, animal use and animal welfare
**Red meat animal rights**	4.17	0.00	**3.99**	**3.73**	**0.24**	0.26	CATI respondents have a more positive attitude toward red meat (beef cattle and sheep) animal rights
**Public engagement beliefs**	4.50	0.00	**3.52**	**3.22**	**0.30**	0.28	CATI respondents have a more positive attitude toward public engagement (i.e., lobbying government, supporting animal welfare causes, and animal welfare promotion)
**Negative normative beliefs**	−4.25	0.00	**2.89**	**3.17**	−**0.28**	−0.26	PANEL respondents have a greater belief that relevant others would not expect them to show public engagement (i.e., lobbying government, supporting animal welfare causes, and animal welfare promotion)
**Positive normative beliefs**	4.62	0.00	**3.30**	**3.00**	**0.30**	0.29	CATI respondents have a greater belief that relevant others would expect them to show public engagement (i.e., lobbying government, supporting animal welfare causes, and animal welfare promotion)
**Easy to act**	−3.92	0.00	**2.81**	**3.05**	−**0.24**	−0.24	CATI respondents see greater ease in supporting or promoting positive animal welfare
**Difficult to act**	3.16	0.00	**3.13**	**2.91**	**0.22**	0.20	PANEL respondents see greater difficulty in supporting or promoting positive animal welfare
Trust	−1.20	0.23	3.38	3.46	−0.08	−0.07	No significant difference in CATI and PANEL respondents’ trust of farmers and animal handlers to appropriately care for beef cattle and sheep
**Approval of husbandry practices**	2.36	0.02	**3.04**	**2.92**	**0.12**	0.15	CATI respondents have a more positive attitude toward the husbandry practices used in the red meat industry
**General welfare**	9.01	0.00	**4.77**	**4.43**	**0.34**	0.56	CATI respondents have a greater belief that good animal welfare requires a range of different factors to be met
**Medication**	6.10	0.00	**4.55**	**4.25**	**0.30**	0.38	CATI respondents have a greater belief that it is important to provide medication to beef cattle and sheep
**Land beef transport conditions**	−4.47	0.00	**2.50**	**2.82**	−**0.32**	−0.28	PANEL respondents have a more positive attitude toward land transport conditions for beef cattle
**Sea beef transport conditions**	−6.53	0.00	**2.11**	**2.57**	−**0.46**	−0.41	PANEL respondents have a more positive attitude toward sea transport conditions for beef cattle
**Land sheep transport conditions**	−4.11	0.00	**2.36**	**2.65**	−**0.29**	−0.26	PANEL respondents have a more positive attitude toward land transport conditions for sheep
**Sea sheep transport conditions**	−6.23	0.00	**2.03**	**2.47**	−**0.44**	−0.39	PANEL respondents have a more positive attitude toward sea transport conditions for sheep
Commercial media	0.53	0.59	2.02	1.99	0.03	0.03	No significant difference in CATI and PANEL respondents’ attitude toward commercial media as a source of knowledge
**Social and internet media**	5.10	0.00	**2.73**	**2.42**	**0.31**	0.32	CATI respondents have a more positive attitude toward social and internet media as a source of knowledge
**Conventional media**	4.99	0.00	**2.61**	**2.33**	**0.28**	0.31	CATI respondents have a more positive attitude toward conventional media as a source of knowledge
**Eats meat**	2.59	0.01	**1.11**	**1.06**	**0.05**	0.16	CATI respondents are more likely to be a vegetarian or vegan
Trust conventional media	−1.04	0.30	3.02	3.08	−0.06	−0.06	No significant difference in CATI and PANEL respondents’ trust of conventional media
**Trust Commercial media**	−4.86	0.00	**2.61**	**2.85**	−**0.24**	−0.30	PANEL respondents are more trusting of commercial media
**Trust social and internet media**	−3.51	0.00	**2.96**	**3.14**	−**0.18**	−0.22	PANEL respondents are more trusting of social and internet media
**Perceived knowledge of beef cattle production**	−0.55	0.00	**2.80**	**3.25**	−**0.45**	−0.41	PANEL respondents have a greater perceived knowledge of beef cattle production
**Perceived knowledge of sheep production**	−5.07	0.00	**2.93**	**3.29**	−**0.36**	−0.32	PANEL respondents have a greater perceived knowledge of sheep production
Knowledge Score	0.68	0.50	72.25	71.46	0.79	0.04	No significant difference in CATI and PANEL respondents’ knowledge score
**Behavior**	2.41	0.02	**2.00**	**1.72**	**0.28**	0.15	CATI respondents perform more community behaviors
**During the past 6 months, how many people have you told about farm animal welfare in Australia?**	**4.71**	**0.00**	**2.34**	**1.95**	**0.39**	0.29	CATI respondents perform more communication activities
**Compared with your friends, how likely are you to be asked about farm animal welfare in Australia?**	**3.50**	**0.00**	**2.25**	**1.98**	**0.27**	0.22	CATI respondents perform more communication activities
Overall, in all of your discussions with friends and neighbors how often are you used as a source of advice on farm animal welfare in Australia?	1.48	0.14	1.88	1.78	0.10	0.09	No significant difference in CATI and PANEL respondents being used as a source of advice on farm animal welfare

In general, CATI respondents were more engaged in community behaviors when compared with the PANEL sample, with significantly more respondents having posted/shared information about an issue on social media, signed a petition and spoken to colleagues, family members or friends in opposition of beef cattle and sheep farming (Table 5).

**TABLE 5:** Independent 2-tailed *t*-tests (df = 1029) comparing engagement with individual community behaviors between the CATI survey and the PANEL survey respondents with education as a covariate.

			Mean				
	t	Sig.	CATI	PANEL	Mean Difference (CATI-PANEL)	Cohen’s D	Interpretation
Written a letter to a politician	−1.68	0.09	0.06	0.09	−0.03	−0.10	No significant difference between CATI and PANEL respondent’s prevalence of writing a letter to a politician
**Posted/shared information about an issue on social media**	4.37	0.00	**0.35**	**0.23**	**0.12**	0.27	CATI respondents post or share more information about an issue on social media
**Called a radio talk back segment**	−3.95	0.00	**0.01**	**0.05**	−**0.04**	−0.25	PANEL respondents called a radio talk back segment more frequently
Attended a public rally or demonstration	0.02	0.98	0.07	0.07	0.00	0.00	No significant difference in CATI and PANEL respondents’ attendance at public rally or demonstration
**Signed a petition**	1.94	0.05	**0.40**	**0.34**	**0.06**	0.12	CATI respondents sign petitions more frequently
**Donated money to animal welfare organizations**	2.20	0.03	**0.48**	**0.41**	**0.07**	0.14	CATI respondents donate money to animal welfare organizations more frequently
Donated goods other than money to animal welfare organizations	−0.33	0.74	0.24	0.25	−0.01	−0.02	No significant difference in CATI and PANEL respondents’ donation of goods other than money to animal welfare organizations
Volunteered your services to animal welfare organizations	−0.20	0.84	0.12	0.12	0.00	−0.01	No significant difference in CATI and PANEL respondents’ volunteering their services to anima welfare organizations
**Spoken to colleagues, family members, or friends**	7.11	0.00	**0.66**	**0.45**	**0.21**	0.44	CATI respondents speak with colleagues, family members and friends about animal welfare more frequently
**Written a letter to a newspaper**	−3.15	0.00	**0.02**	**0.05**	−**0.03**	−0.20	PANEL respondents have a greater prevalence of writing a letter to a newspaper

There was no significant difference between CATI and PANEL respondents in relation to the regularity of their red meat consumption (Table 6).

**TABLE 6:** Independent 2-tailed *t*-tests (df = 1029) comparing the consumer behavior of the CATI survey and the PANEL survey respondents with education as a covariate.

			Mean				
	t	Sig.	CATI	PANEL	Mean Difference (CATI-PANEL)	Cohen’s D	Interpretation
How often would you eat beef in an average week?	−0.53	0.59	3.30	3.34	−0.04	0.03	No significant difference in CATI and PANEL respondents’ average weekly beef consumption
How often would you eat lamb in an average week?	0.45	0.65	2.40	2.37	0.03	0.03	No significant difference in CATI and PANEL respondents’ average weekly lamb consumption

### Comparing CATI and PANEL Samples: Relationships Between Attitudes, Knowledge and Community Behavior

Correlations amongst the composite attitude variables and the four outcome variables (actual knowledge, community behavior and both perceived knowledge scores) are given in Table 7. For the correlations between attitudes and actual knowledge, in every case where significant differences exist between the two survey samples, the PANEL correlations are significantly larger than the CATI correlations. With one exception, this is also the case for correlations between attitudes and perceived knowledge for both beef cattle and sheep.

**TABLE 7:** Correlations (df = 1030) between Knowledge, Community behavior and all composite variables.

	Actual Knowledge		Behavior		Perceived Knowledge beef		Perceived Knowledge sheep	
	CATI	PANEL	CATI	PANEL	CATI	PANEL	CATI	PANEL
Animal welfare humane	**0.03**	**0.29**	0.19	0.20	−0.05	0.00	−0.01	−0.01
Animal welfare handling	**0.12**	**0.25**	0.01	0.11	−0.09	−0.10	−0.04	−0.10
Animal welfare people animals	**0.00**	**0.17**	0.13	0.11	−0.02	−0.14	0.00	−0.13
Red meat attributes	0.08	0.10	−0.34	−0.20	−0.15	−0.04	−0.15	−0.03
Red meat animal rights	−0.04	0.05	0.27	0.24	0.04	−0.04	0.01	−0.05
Public engagement beliefs	−0.15	−0.08	0.51	0.49	**0.03**	−**0.16**	**0.02**	−**0.16**
Negative normative beliefs	0.08	−0.03	−**0.35**	−**0.15**	0.05	0.12	0.04	0.14
Positive normative beliefs	−0.11	−0.07	0.41	0.44	−**0.04**	−**0.23**	−**0.09**	−**0.22**
Easy to act	−0.08	−0.17	−0.19	−0.14	0.15	0.19	0.19	0.20
Difficult to act	−0.09	−0.13	0.44	0.42	−0.12	−0.21	−0.06	−0.20
Trust	0.02	−0.05	−**0.39**	−**0.21**	−0.13	−0.02	−0.14	−0.01
Approval of husbandry practices	0.17	0.05	−**0.31**	−**0.07**	−0.24	−0.16	−0.19	−0.14
General welfare	**0.03**	**0.30**	**0.28**	**0.09**	0.04	−0.05	0.05	−0.07
Medication	0.01	0.15	0.04	0.06	0.04	−0.08	0.02	−0.08
Land beef transport conditions	−**0.03**	−**0.20**	−**0.32**	−**0.16**	−0.16	−0.04	−**0.18**	−**0.02**
Sea beef transport conditions	−**0.01**	−**0.27**	−**0.35**	−**0.16**	−0.10	−0.05	−0.09	−0.01
Land sheep transport conditions	−**0.02**	−**0.23**	−**0.33**	−**0.19**	−0.12	−0.06	−0.14	−0.02
Sea sheep transport conditions	**0.00**	−**0.28**	−**0.35**	−**0.18**	−0.08	−0.05	−0.07	−0.01
Commercial media	−0.06	−0.15	**0.20**	**0.48**	−**0.14**	−**0.27**	−**0.14**	−**0.27**
Social and internet media	−0.09	−0.04	**0.53**	**0.64**	−**0.02**	−**0.27**	**0.03**	−**0.25**
Conventional media	0.13	0.01	**0.15**	**0.47**	−**0.19**	−**0.32**	−0.24	−0.32
Trust conventional media	−0.07	−0.03	0.14	0.22	0.10	−0.01	0.11	−0.01
Trust Commercial media	−0.15	−0.15	0.05	0.15	0.12	0.00	0.07	0.02
Trust social and internet media	−0.17	−0.05	0.39	0.40	0.10	−0.06	0.09	−0.04

*Pairs of correlations in bold are significantly different at *p* < 0.05.*

Correlations between attitudes and behavior show the opposite pattern to the knowledge and perceived knowledge correlations. In most instances where significant differences exist between the two survey samples, the PANEL correlations are significantly larger than the CATI correlations. However, where the correlations are between accessing the three media types (commercial, conventional and social) and behavior, the correlations are significantly larger for the CATI sample.

When all composite variables were entered into a linear regression model with community behavior as the dependent variable, five variables uniquely contributed to predicting community behavior for respondents of the CATI survey (Public engagement beliefs, Positive normative beliefs, Trust, Social and internet media and Eats meat) and accounted for 47% of its variance (Table 8). For the PANEL sample, five variables uniquely contributed to predicting Community behavior (Public engagement beliefs, Commercial media, Social and internet media, Eats meat and Trust Commercial media) and these variables accounted for 48% of its variance (Table 9).

**TABLE 8:** Linear regression with Community behavior as the dependent variable and all composite variables entered as the predictors, for the CATI sample.

	Beta coefficient (standardized)	t	Sig.
(Constant)		−2.0	0.04
Public engagement beliefs	0.15	2.72	0.01
Positive normative beliefs	0.13	2.73	0.01
Trust	−0.17	−3.50	0.00
Social and internet media	0.32	7.10	0.00
Eats meat	0.10	2.50	0.01

**R*^2^ = 0.47*

**TABLE 9:** Linear regression with Community behavior as the dependent variable and all composite variables entered listwise as the predictors, for the PANEL sample.

	Beta coefficient (standardized)	t	Sig.
(Constant)		−3.52	0.00
Public engagement beliefs	0.18	2.86	0.00
Commercial media	0.15	2.69	0.01
Social and internet media	0.35	5.78	0.00
Eats meat	0.07	2.07	0.04
Trust commercial media	−0.11	−2.20	0.03

**R*^2^ = 0.48.*

### Comparing CATI and PANEL Samples: Relationships Between Attitudes and Red Meat Consumption

Correlations between the composite attitude variables and self-reported frequency of beef and lamb consumption are given in Table 10. In every case where significant differences in the correlations exist between the two survey samples, the CATI correlations are significantly larger than the PANEL correlations.

**TABLE 10:** Correlations (df = 1030) between beef and lamb consumption and all composite variables.

	Beef		Lamb	
	CATI	PANEL	CATI	PANEL
Animal welfare humane	−**0.16**	**0.02**	–0.11	0.01
Animal welfare handling	0.08	0.10	0.07	0.09
Animal welfare people animals	–0.04	0.07	0.00	0.04
Red meat attributes	**0.55**	**0.38**	**0.48**	**0.29**
Red meat animal rights	–0.22	–0.14	–0.16	–0.04
Public engagement beliefs	−**0.26**	−**0.12**	–0.12	0.01
Negative normative beliefs	0.22	0.15	**0.16**	**0.03**
Positive normative beliefs	–0.11	–0.03	–0.05	0.07
Easy to act	0.01	0.03	0.06	–0.03
Difficult to act	–0.18	–0.06	–0.15	0.06
Trust	**0.48**	**0.28**	0.34	0.23
Approval of husbandry practices	**0.41**	**0.13**	**0.33**	**0.15**
General welfare	−**0.16**	**0.01**	–0.12	–0.02
Medication	0.12	0.08	0.10	0.00
Land beef transport conditions	**0.40**	**0.15**	**0.33**	**0.09**
Sea beef transport conditions	**0.34**	**0.14**	**0.28**	**0.10**
Land sheep transport conditions	**0.40**	**0.18**	**0.33**	**0.09**
Sea sheep transport conditions	**0.32**	**0.14**	**0.25**	**0.11**
Commercial media	0.04	0.01	0.07	0.09
Social and internet media	–0.22	–0.12	−**0.18**	−**0.02**
Conventional media	0.01	0.01	–0.01	0.11
Trust conventional media	–0.08	–0.05	–0.04	0.06
Trust Commercial media	0.11	–0.01	0.11	0.13
Trust social and internet media	–0.24	–0.11	–0.15	0.01

*Pairs of correlations in bold are significantly different at *p* < 0.05.*

When all composite variables were entered into linear regression models with self-reported consumption of beef as the dependent variable, two variables uniquely contributed to predicting beef consumption (Red meat attributes and Trust) for respondents of the CATI survey and accounted for 38% of its variance (Table 11). For the PANEL sample, five variables uniquely contributed to predicting beef consumption (Red meat attributes, Red meat animal rights, Negative normative beliefs, Approval of husbandry practices and Medication) and these variables accounted for 19% of its variance (Table 11). Unlike the analysis of behavior, a much smaller percentage of the variance in beef consumption was predicted in the PANEL sample compared to the CATI sample.

**TABLE 11:** Linear regression with beef consumption or lamb consumption as the dependent variable and all composite variables entered listwise as the predictors.

Beef consumption		Beta coefficient (standardized)	t	Sig.	R^2^
CATI	(Constant)		2.19	0.03	0.38
	Red meat attributes	0.36	6.62	0.00	
	Trust	0.13	2.48	0.01	
PANEL	(Constant)		4.39	0.00	0.19
	Red meat attributes	0.36	6.20	0.00	
	Red meat animal rights	−0.14	−2.80	0.00	
	Negative normative beliefs	0.10	2.07	0.04	
	Approval of husbandry practices	−0.11	−2.30	0.03	
	Medication	0.12	2.01	0.04	
**Lamb consumption**					
CATI	(Constant)		1.22	0.22	0.25
	Red meat attributes	0.41	7.10	0.00	
	Public engagement beliefs	0.16	2.58	0.01	
PANEL	(Constant)		3.22	0.00	0.09
	Red meat attributes	0.25	4.10	0.00	

When all composite variables were entered into linear regression models with self-reported consumption of lamb as the dependent variable, two variables uniquely contributed to predicting lamb consumption for respondents of the CATI survey (Red meat attributes and Public engagement beliefs) and accounted for 25% of its variance (Table 11). For the PANEL sample, only one variable uniquely contributed to predicting lamb consumption (Red meat attributes) and this variable accounted for 9% of its variance (Table 11). Similar to beef consumption, a much smaller percentage of the variance in lamb consumption was predicted in the PANEL sample compared to the CATI sample.

## Discussion

The primary aim in this paper was to determine whether there were differences between the CATI (RDD telephone) and the PANEL (PIP) survey samples in animal welfare-related attitudes and behavior, and the interrelationships amongst these variables. As a first step, it was important to compare the two samples in demographic characteristics. In some respects, it is not surprising that the results of this study showed that the demographic characteristics of the two samples were quite similar. Quotas were applied to age and gender and the data showed the two samples to be similar in these respects. However, geographical distributions which had not been sampled by quota were also similar to both each other and also to the Australian census data. The one demographic variable where the survey samples differed from each other was in education. It is not clear why there were fewer technical and further education educated people in the CATI sample and (although not significant) more university or other higher educational institution educated people compared to the PANEL sample. Given these differences, we accounted for the potential impact of education in analysis of the data by adjusting for level of education achieved.

A second point of comparison that will assist in interpreting the similarities and differences between the samples is response rate. Response rates for internet and telephone surveys consistently show that telephone surveys take longer to complete than do internet surveys. As indicated earlier, Ansolabehere and Schaffner (2014) compared internet and telephone surveys and found response rates of 42.9% and 19.5%, respectively. Similarly, Link and Mokdad (2005) reported response rates of 40.1% and 15.4%, respectively. In the current study the response rates were 10% and 15%, respectively. It is not clear why the differences in response rates between this study and previous studies occurred.

For the telephone survey, Ansolabehere and Schaffner (2014) reported a response rate of 20.9% for landline telephone numbers and 8.6% for mobile telephone numbers. They received 807 landline and 100 mobile telephone responses. If these numbers are recalculated for a sample consisting of a 50:50 split of landline and mobile telephone respondents, the average response time for all telephone contacts in Ansolabehere and Schaffner’s (2014) study would be 14.8% compared to 15% in the current study and 40.1% in the study by Link and Mokdad (2005). However, the methodology used by Link and Mokdad (2005) was quite different from both Ansolabehere and Schaffner (2014) and the current study because telephone respondents first received the questionnaire via mail and were then contacted by telephone. Thus, it appears that the response rate for telephone respondents in the current study are similar to those obtained by at least one other study that used similar methodology.

For the PANEL survey, the response rate in the current study is much lower than that obtained by Ansolabehere and Schaffner (2014), however, it is difficult to compare the methodologies used in the two studies. Ansolabehere and Schaffner (2014) used a procedure where everyone in the target sample as defined by census data is matched with at least one person from the internet panel. The survey link is then sent to the selected panelists and the responses are weighted to ensure that the matched sample is representative of the population target sample.

Given the length of the questionnaire used in the current study (CATI: 33.3 min, PANEL: 19 min) it might be expected that the response rates would be lower than that obtained in other studies. For example, completion times in Ansolabehere and Schaffner’s (2014) study were 14.3 min for telephone respondents and 8.9 min for internet respondents.

Although the underlying explanation for the differences between the studies is unclear, it seems reasonable that the two samples used in the current study should be comparable in terms of representativeness because of similarities in the demographic data, consistency with census data except for education, and similar response rates, even if they do not correspond to those obtained in other studies.

A comparison of the CATI sample with the PANEL sample on the composite variables showed that the PANEL sample gave generally more conservative responses than did the CATI sample, in the sense that they were more positive toward the red meat industry and animal welfare within these industries. In evaluating these differences between the two samples, it is important to consider their practical importance. The analysis of the demographic factor of education level that differed between the two samples showed an effect size of a partial ^2^η = 0.10 compared with an effect size associated with the substantive dependent variables of ^2^η = 0.28. While the sample difference in education is substantial, the differences in the substantive variables after controlling for education is quite large. Further, many of the univariate effect sizes are in the small to medium range. While care needs to be taken not to interpret sample differences in each of the dependent variables in isolation because of the multiple tests carried out with the consequent increase in Type I error rate, the multivariate test and the overall pattern of univariate results suggest that there is a meaningful difference between the two samples in many of the attitude and behavior measures.

It is unclear why the PANEL sample gave what appear to be generally more conservative responses than did the CATI sample. There are few studies that have investigated the relationship between survey type and conservatism. Ansolabehere and Schaffner (2014) in their study of US respondents, reported that, compared to telephone survey respondents, internet respondents were more likely to say, for example, that budget cuts should come more from defense spending. Further, the internet survey produced much lower estimates of the proportion of the population that were supportive of Congress and of affirmative action. It is difficult to infer the orientation underpinning these response patterns; results regarding support for affirmative action suggest that internet survey respondents may be more conservative than telephone survey respondents. However, support for budget cuts coming from cuts to defense spending and lack of support for Congress seem to imply the opposite orientation. It also may be that the political view reflected in these responses are not indicators of the same sort of conservatism that is reflected in the responses observed in the current study.

It may be that people who have agreed to participate in surveys on a regular basis (the PANEL sample), who are recorded on a register by the market research company and who receive a reward for participating, may be more susceptible to socially desirable responses than those who have responded to a “cold call” on a telephone (the CATI sample). There are no data from this study to support this and it is a different conclusion to that reached by Lee et al. (2015) who concluded that, when the subject matter was gambling and its co-morbidities (alcohol, drug and tobacco use), online responses were less susceptible to social desirability because the online survey ensured greater anonymity. Further, Vesely and Klöckner (2020) found little evidence for social desirability to be a confounder of people’s survey responses regarding environmental actions, and, to the extent that animal welfare attitudes might align with attitudes toward environmental action, this might apply to the results reported here. However, this is a speculative argument and requires empirical investigation to determine its relevance or validity. If anonymity is the determining factor, then the results of Lee et al. (2015) are consistent with those found here, because in the current study it was the CATI survey that provided greater anonymity.

Differences between the two survey samples in response distributions is important if the prevalence of attitudes, knowledge and behavior is of interest. This is because it is the prevalence of these measures of the public and/or consumers that may inform responses by legislators, welfare groups and industry to people’s attitudes. It would be worthwhile, therefore, to establish the reliability of the differences observed here by replicating the study and to establish, through the inclusion of some follow-up questions, whether perceived anonymity was the factor that led to the observed differences.

In addition to differences in distributions of responses, it is important to establish that the importance of the attitudes in relation to people’s behavior is similar in the two samples. If one sample is more compliant, it may be that respondents’ attitudes are less related to their actions than in a sample where responses are less susceptible to social desirability. In fact, where significant differences in correlations were found, the PANEL sample showed higher correlations with knowledge scores, but lower correlations with community and consumption behaviors when compared to the CATI sample. This is consistent with the hypothesis that where self-reported subjective matters are involved, the PANEL sample responds consistently with a social desirability bias. On the other hand, where attitudes are correlated with a self-reported objective behavior, the stronger correlations occur in the CATI sample which may be less susceptible to social desirability bias. There is a clear speculative element to this argument, and it needs to be tested in a study that identifies the factors discussed in the previous paragraph.

The differences in correlations between the composite variables and meat consumption were also reflected in the amount of variance in consumption that was predicted in the two samples. The PANEL sample showed consistently lower prediction of consumption than did the CATI sample. Not only were demographics similar for the two samples, so were beef and lamb consumption. The differences that occurred in predicting consumption between the two samples are not related to actual consumption. It is unclear why the two samples differed in the prediction of consumption; it is difficult to attribute it to overall differences in attitudes to the livestock industries.

Berrens et al. (2003) compared RDD telephone surveys with PIP surveys on the issue of global climate change and found that the internet sample produced relational inferences similar to that of the telephone sample despite many differences also existing. In their study it was unclear whether it was possible to identify characteristics of respondents from the internet sample. Also, it may be that attitudes to climate change are less susceptible to factors associated with the data collection method than animal welfare attitudes and associated behaviors. Berrens et al. (2003) suggest that, in addition to offering a viable means of data collection, PIP surveys also provide advantages over RDD telephone surveys in terms of response quality. Respondents’ previous experience completing surveys is believed to be the reason for increased response quality among the internet sample when compared to the telephone sample. In contrast to the findings by Berrens et al. (2003), studies such as those by Flemming and Sonner (1999) and Taylor (2000) and the review by Couper (2000) report significant differences between RDD telephone and PIP survey samples regarding both background characteristics as well as other research variables, including attitude items, health indicators, and voting tendencies. Flemming and Sonner (1999) suggest that a lack of predictable patterns to the differences found between the survey approaches raises important questions about the viability of PIP surveys to replace RDD telephone surveys. Furthermore, Lee et al. (2015) and Couper (2000) suggest that while some of the differences lessen with propensity weighting, other differences between the samples remain unaffected by such weighting. It is clear that further investigation of the psychological variables that systematically differ between samples collected using different survey methodologies is needed.

Interestingly, despite the differences in prevalence of community behaviors, the composite scores predicted a similar proportion of the variance in such behavior in both samples in the current study. There was some commonality in the predictors of community behavior (e.g., Public engagement beliefs and Eats meat), but the other predictors differed between the samples, consistent with the differing patterns of correlations. This suggests that attitudes generally are strong predictors of community behavior even if the salient beliefs differ. This is consistent with the findings of Coleman et al. (2017) who found that attitude variables in a CATI sample of Australians accounted for 44% of the variance in community behavior. The implications of this for the current study are that there is a robust relationship between attitudes and community behavior, regardless of the sampling technique employed. However, care needs to be taken when interpreting such results in terms of which attitude is the most important driver.

In addition to social desirability, factors such as internet access may account for some of the differences found between RDD telephone and PIP survey samples. However, the differences may also relate to prosocial behavior, i.e., survey participation (taking time to complete a survey that does not directly benefit them). Like volunteering, participation in surveys is a form of prosocial behavior (Bekkers, 2012). Whilst the reason why a person might choose to participate in a PIP is likely to involve the financial compensation, it may also involve an element of volunteerism. If this is the case, volunteerism will be associated with any form of survey participant, however, this may be greater in PIPs because they have volunteered for ongoing survey participation. If volunteerism does involve a prosocial element, then it may be that they are more susceptible to social desirability, consistent with results indicating PANEL respondents reporting less concern about the red meat industry and animal welfare in comparison to the respondents from the RDD telephone survey (CATI sample). Therefore, in this case a PIP survey may underestimate concern about red meat farming in Australia in the general population. Given the increased use of PIP surveys, the reasoning behind this type of ongoing online panel participation and its impact on survey results requires further investigation.

Furthermore, if research is aimed at assessing the prevalence of public attitudes toward animal welfare issues, a PIP survey may not be the most appropriate method. It is not clear why the PIP survey sample is more conservative with regard to animal use and animal welfare in the red meat industry when compared to the RDD telephone survey sample, and it may be useful to explore this further because of the implications for interpreting the results from other kinds of surveys.

## Conclusion

We found differences between the two survey samples in both attitudes and behavior toward the red meat industry. The PANEL respondents generally gave more conservative responses than did the CATI respondents in that they were more positive toward the red meat industry and animal welfare within these industries. This was also reflected in behavior relating to the red meat industry, both community and consumer behavior. Thus, a PIP survey may underestimate concern about red meat farming in Australia in the general population. However, further research is required to identify the specific psychological factors that underpin the differences between samples derived from the different survey methodologies.

## Data Availability Statement

The datasets presented in this article are not readily available because: Our ethics approval specifies that “The survey results will only be reported for groups, so individual responses cannot be identified”. Therefore, we cannot supply the raw data even if it has been anonymised without special approval from the ethics committee via an amendment. Requests to access the datasets should be directed to LH, lauren.hemsworth@unimelb.edu.au.

## Ethics Statement

The studies involving human participants were reviewed and approved by the Human Ethics Advisory Group, The University of Melbourne Ethics ID: 1750676.3. Informed consent for participation was required for both the CATI (verbal consent given after PLS statement read) and the PANEL (after reading PLS statement participant completing the questionnaire was taken as inferred consent).

## Author Contributions

LH, MR, PH, and GC were responsible for conceptualization and design. Data analysis was performed by LH, MR, and GC. LH drafted the manuscript and it was reviewed and edited by all authors.

## Conflict of Interest

The authors declare that this study received funding from Meat & Livestock Australia (MLA) and the University of Melbourne. The funders were not involved in the study design, collection, analysis, interpretation of data, the writing of this article or the decision to submit it for publication.
